# Serum withdrawal establishes a stress-dominant entry state during myogenic differentiation

**DOI:** 10.3389/fcell.2026.1823860

**Published:** 2026-05-25

**Authors:** Zongnan Lyu, Chunxue Shao, Renyu Yang, Qi Yu, Minzhuo Yang, Guang Yang, Ziheng Wang

**Affiliations:** 1 Department of Sports Science, Zhejiang University, Hangzhou, China; 2 Division of Computational Biology, Chinese Center of Exercise Epidemiology, Northeast Normal University, Changchun, China

**Keywords:** C2C12 differentiation, cell-cycle exit, serum withdrawal, skeletal myogenesis, stress-associated transcription, transcriptomic reanalysis

## Abstract

**Introduction:**

Skeletal myogenesis in C2C12 cultures is typically induced by switching confluent cells from high-serum growth medium to low-serum differentiation medium. Because serum withdrawal is itself an acute physiological perturbation, early post-switch transcriptional changes may reflect varying mixtures of lineage progression and stress adaptation.

**Methods:**

Here we performed a descriptive reanalysis of publicly available bulk RNA-seq datasets spanning (i) canonical C2C12 differentiation by serum withdrawal, (ii) bovine satellite-cell differentiation under serum-withdrawal versus serum-free or chemically defined conditions, (iii) additional bovine serum-free or defined-condition validation datasets, and (iv) a non-canonical serum-free \textit{Myod1} loss-of-function perturbation. Using a unified rank-based framework, we quantified stress-associated transcription, myogenic programme activation, and cell-cycle exit, and summarised their relationships using composite axes of stress dominance, transition abruptness, and exit--myogenic alignment.

**Results:**

Across bovine and murine serum-withdrawal time-course datasets, the earliest post-induction states showed relatively elevated and temporally leading stress-associated scores, together with weaker alignment between myogenic activation and durable cell-cycle withdrawal; by contrast, non-withdrawal conditions showed more gradual trajectories and stronger alignment. Independent bovine validation datasets provided directionally consistent external support for this descriptive contrast, showing progressive myogenic organisation and exit-related structure under non-withdrawal conditions or enhanced media without selective expansion of stress-dominant states. In the non-canonical serum-free \textit{Myod1} perturbation, myogenic scores decreased and cell-cycle-related scores shifted, whereas stress-associated scores remained similar, indicating a descriptive dissociation between these axes in a restricted perturbational context rather than evidence of physiological differentiation dynamics.

**Discussion:**

Together, these analyses provide operational, structure-neutral criteria for comparing induction conditions and interpreting early myogenic differentiation readouts across protocols.

## Graphical Abstract

**Figure:**
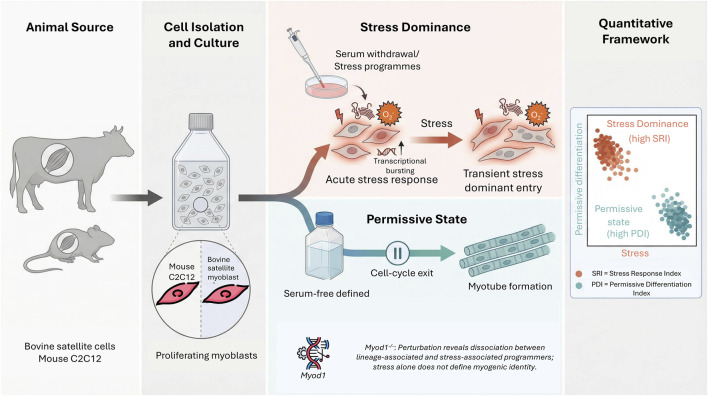

## Highlights

• Serum withdrawal induces an early stress-dominant transcriptional state.

• Non-withdrawal and chemically defined conditions show smoother transitions with stronger exit–myogenic alignment.

• A unified rank-based framework quantifies stress dominance, transition abruptness, and myogenic progression across protocols.

## 1 Introduction

Skeletal myogenesis is a core model for studying cell-fate commitment, transcriptional control, and tissue regeneration, because myoblasts pass through a stereotyped sequence of cell-cycle withdrawal, lineage-gene activation, and fusion into multinucleated myotubes (Dumont et al., 2015; Chal and Pourquié, 2017; Rajan et al., 2012). The murine C2C12 line, derived from the Yaffe and Saxel myogenic lineage, is one of the most widely used *in vitro* systems for mechanistic work in this field (Yaffe and Saxel, 1977; Rajan et al., 2012; Tannu et al., 2004). In standard practice, differentiation is initiated by switching confluent cultures from high-serum growth medium to low-serum medium (typically DMEM with 2% horse serum), and early post-switch samples are commonly interpreted as the onset of the myogenic programme (Taye et al., 2020; Fujita et al., 2010; Andrés and Walsh, 1996; Messmer et al., 2022).

This interpretation has an important caveat: the medium switch is not only a temporal trigger, but also an acute physiological perturbation. Serum supplies growth factors, hormones, lipids, and carrier proteins that shape insulin and insulin-like growth factor (IGF) signalling, nutrient sensing, and survival pathways; its withdrawal therefore abruptly changes trophic input (Párrizas et al., 1997; Khan et al., 2025). Loss of IGF-linked pro-survival signalling can amplify stress-related and apoptotic programmes (Novosyadlyy et al., 2008), and serum deprivation is known to induce adaptive responses that include stress-kinase activation and nutrient-deprivation transcriptional programmes (Gama Sosa et al., 2016; Valverde et al., 2004; Costa-Mattioli and Walter, 2020). As a result, early transcriptional changes after induction are intrinsically composite, potentially reflecting both lineage progression and environmental adaptation.

This conflation of early myogenic progression with acute serum-withdrawal-induced stress responses may contribute to a persistent practical problem: limited agreement across studies on the timing of marker transitions and pathway dependencies during C2C12 differentiation (Trapnell et al., 2014). Such discrepancies are often attributed to technical variation, yet protocols that avoid abrupt withdrawal—that is, non-withdrawal conditions such as serum-free and chemically defined conditions—frequently show reduced stress signatures, altered kinetics of canonical myogenic regulators, and distinct metabolic trajectories (Messmer et al., 2022). These observations imply that transcriptional states labelled as “differentiating” are not necessarily equivalent across induction conditions.

The distinction is consequential. *In vivo* muscle repair requires coordinated cell-cycle exit, activation of myogenic programmes, and bioenergetic remodelling within a supportive niche (Ryall et al., 2015). *In vitro*, by contrast, early samples may be enriched for immediate-early or stress-associated modules alongside myogenic and exit-associated programmes, complicating interpretation of early “differentiation markers” and short-window causal inference (García-Prat et al., 2016). In translational settings—including drug screening, regenerative bioprocessing, and cultured-meat production—treating these early composite states as interchangeable with later maturation-associated states risks misjudging product quality and intervention efficacy (Post et al., 2020; Chal and Pourquié, 2017).

Here we introduce an operational, structure-neutral framework to summarise composite transcriptional organisation across *in vitro* myogenesis protocols. By reanalysing bulk RNA-seq trajectories from serum-withdrawal and non-withdrawal conditions in mouse and bovine systems, together with additional bovine validation datasets and a serum-free *Myod1* loss-of-function perturbation, we quantify stress-associated transcription, myogenic activation, and cell-cycle exit using a unified rank-based scoring strategy. This framework enables direct comparison of condition-dependent programme weighting, temporal offset, and coupling, and provides a principled basis for interpreting early differentiation readouts without assuming mechanistic separability.

## 2 Methods

We reanalysed publicly available transcriptomic datasets spanning (i) canonical C2C12 differentiation induced by serum withdrawal, (ii) bovine satellite-cell differentiation under serum-withdrawal and non-withdrawal conditions (including serum-free and chemically defined settings), (iii) additional bovine validation datasets used to test whether the same descriptive framework generalises beyond the primary comparisons, and (iv) a serum-free *Myod1* loss-of-function perturbation in C2C12 cells. All datasets were processed through a single analysis workflow that quantifies stress-associated transcription, myogenic progression, and cell-cycle state using rank-based gene-module scores and derived composite programme axes.

### 2.1 Study scope and inferential framework

This study is a reanalysis of public bulk RNA-seq data and is therefore designed for *descriptive* inference rather than mechanistic attribution. We use *stress-associated state* to denote early post-induction states in which rapid environmental change coincides with quantitatively prominent immediate-early/integrated-stress transcription, and *non-withdrawal conditions* to denote serum-free or chemically defined settings in which myogenic activation and cell-cycle exit evolve under comparatively stable media inputs.

Our primary claim is comparative and testable: across datasets, early post-induction variation can be summarised by a stress-associated axis that may be relatively enriched after medium perturbation and a myogenic-progression axis that changes with commitment markers. We do not assume universal separability between stress programmes and myogenesis; instead, we test whether stress-associated signatures can be relatively enriched at early time points, thereby confounding interpretation when early sampling is treated as a direct proxy for commitment.

To limit over-interpretation, cross-species conclusions are restricted to directionally consistent patterns. We additionally report identifier-mapping yield and module-level missingness after cross-species mapping.

### 2.2 Datasets and study design

We prioritised studies with gene-by-sample matrices to enable uniform processing. Primary bovine satellite-cell data were obtained from the GSE173199 superseries (*Bos taurus*) (Messmer et al., 2022), which comprises the serum-free differentiation subseries GSE173196 and the serum-withdrawal time-course subseries GSE173198. These data included (i) a serum-withdrawal time-course (20%–2% FBS; 0, 24, 48, 72 and 96 h; four biological replicates per time point) and (ii) serum-free differentiation conditions (72 h; three biological replicates per condition). Within GSE173196, the primary paired differentiation labels were $0_{\mathrm{SFGM}}$ / $3_{\mathrm{SFDM}}$ and $0_{GM}$ / $3_{DM}$ , and the original study also included auxiliary day-3 conditions labelled $3_{\mathrm{DMEM}}$ and $3_{\mathrm{SFB}}$ . We retained these auxiliary states as within-dataset reference conditions in the descriptive analyses to help contextualise programme shifts and to partially disentangle protocol-associated stress responses from myogenic progression. For external validation, we additionally analysed a bovine serum-free single-nucleus RNA-seq time-course aggregated to pseudobulk by time point (GSE240556; 0, 24, 48, 72 and 96 h) (Melzener et al., 2024), and an independent bovine bulk RNA-seq study comparing conventional differentiation medium with enhanced media at day 15 together with day-2 baseline states (GSE262675) (Trautmann et al., 2025).

Mouse C2C12 datasets (*Mus musculus*) comprised a canonical serum-withdrawal time-course from GSE224489 (0, 12, 24, 48, 60 and 96 h; three biological replicates per time point) (Zhao et al., 2023) and a serum-free *Myod1* loss-of-function contrast (GSE137507; wild-type versus ${Myod1}^{-/-}$ ; three biological replicates per genotype) (Xu et al., 2021). Importantly, GSE137507 was originally designed under serum-free conditions to assess neurosphere-forming potential rather than canonical myogenic differentiation. Therefore, this dataset does not represent a physiological or standard myogenic trajectory. Accordingly, we used it exclusively as a perturbational stress-test to evaluate whether stress-associated and lineage-associated programme scores remain separable in a non-canonical serum-free context that is not interpretable as a standard myogenic trajectory. The bovine datasets were prioritised because they provide the most direct accessions for comparing serum-free/defined and withdrawal-associated induction logic, whereas the C2C12 datasets were prioritised because C2C12 remains the most widely used murine model of *in vitro* myogenesis. The public GEO datasets reanalysed in this study are summarised in Table 1.

**TABLE 1:** Public GEO datasets reanalysed in this study.

Accession	Species	Model	Experimental conditions	Use in this study
GSE173196	Bovine	Satellite cells	$0_{\mathrm{SFGM}}$ , $3_{\mathrm{SFDM}}$ , $0_{GM}$ , $3_{DM}$ , $3_{\mathrm{DMEM}}$ , $3_{\mathrm{SFB}}$ ; three biological replicates per condition	Serum-free/defined bovine References and within-study medium comparison
GSE173198	Bovine	Satellite cells	Serum-withdrawal time-course (0, 24, 48, 72, 96 h); four biological replicates per time point	Bovine withdrawal dynamics
GSE240556	Bovine	Satellite cells/nuclei	Serum-free time-course (0, 24, 48, 72, 96 h); analysed as pseudobulk time-point profiles	External bovine serum-free trajectory validation
GSE262675	Bovine	Myogenic cultures	Day-2 baseline media and day-15 conventional versus enhanced differentiation media	External bovine defined/optimised-medium validation
GSE224489	Mouse	C2C12 myoblasts	Serum-withdrawal time-course (0, 12, 24, 48, 60, 96 h); three biological replicates per time point	Canonical murine withdrawal dynamics
GSE137507	Mouse	C2C12 myoblasts	Serum-free wild-type versus ${Myod1}^{-/-}$ comparison; three biological replicates per genotype	Serum-free perturbation comparison

### 2.3 Data processing and identifier mapping

When available, GEO-deposited gene-level count matrices were used directly to preserve traceability to source accessions. Sample identifiers were parsed programmatically and reconciled with GEO metadata to assign time points and conditions.

Within each dataset, raw counts were transformed to ${\text{log}}^{2}$ counts per million ( ${\text{log}}_{2}$ CPM) with a pseudocount of 1 as shown in Equation 1:

$${log_{2}CPM}_{g,s}=\log_{2}\left(\frac{10^{6}\;c_{g,s}}{\sum_{g^{\prime }}c_{g^{\prime },s}}+1\right),$$ (1)

where $c_{g,s}$ is the raw count for gene g in sample s (Law et al., 2014). Programme and module scores were computed *within* datasets after library-size normalisation; expression matrices were not merged across studies. This design reduces sensitivity to platform- and depth-related differences. For confirmatory differential-expression analyses, the limma–voom framework was applied when raw integer counts were available; datasets with only non-integer processed values were analysed descriptively.

For GSE262675, GEO-deposited TPM matrices were analysed descriptively after ${\text{log}}_{2}$ (TPM +1 ) transformation. For GSE240556, nuclei were aggregated by time point to generate pseudobulk profiles before scoring, so that the validation analyses entered the downstream framework as one expression profile per sample or time point. In addition, for GSE240556 only, we computed per-nucleus rank-based programme scores and classified nuclei into descriptive state classes using dataset-wide tertiles of the composite stress response score ( ${\text{COMP}}_{\text{STRESS}}$ ), composite myogenic progression score ${\text{COMP}}_{\text{STRESS}}$ , ${\text{COMP}}_{\text{MYOGENIC}}$ , and Exit. Specifically, “high” was defined as values in the upper tertile of the corresponding score distribution and “low” as values in the lower tertile. Each nucleus was assigned to one mutually exclusive class: stress high, myogenic high, exit high, stress low, myogenic low, exit low, or intermediate mix. When a nucleus satisfied more than one high/low criterion, it was assigned to the class corresponding to the largest absolute deviation from the dataset-wide median of the relevant composite score; nuclei satisfying no high/low criterion were labelled intermediate mix. The class labels were used only as descriptive summaries of relative state composition and were not intended as discrete biological cell types. These supplementary datasets were used to test directional concordance of programme organisation rather than to redefine the primary within-study contrasts. For cross-study comparisons, bovine Ensembl identifiers were mapped to gene symbols using BioMart (Smedley et al., 2015), with fallback parsing of the ARS-UCD1.2 GT F annotation (Ensembl release 110) (Martin et al., 2023). Mappings were cached for reproducible reruns. Mapping yield was recorded globally and per module, and robustness was checked by restricting analyses to unambiguous mappings (including 1:1 orthologue subsets where applicable).

### 2.4 Rank-based module scoring and composite programmes

To quantify programme activity robustly across heterogeneous expression scales, we used a deterministic, rank-based single-sample scoring procedure without gene-level or module-level weighting. Programme and module scores were computed as mean rescaled within-sample gene ranks, with no additional weighting, scaling, or variance adjustment; all computations were performed independently within each dataset to avoid cross-study normalisation effects. For each sample s , genes were ranked by normalised expression ( ${\text{log}}_{2}$ CPM), generating within-sample ranks $r_{g,s}\in \left\{0,\ldots ,G-1\right\}$ . Ranks were then rescaled to the unit interval as shown in Equation 2:

$$R_{g,s}=\frac{r_{g,s}}{G-1}\in \left[0,1\right].$$ (2)

For a predefined gene module M the module score in sample s was defined as the arithmetic mean of the rescaled ranks of retained module genes as shown in Equation 3:

$$Score\left(M,s\right)=\frac{1}{\left|M^{\prime }\right|}\underset{g\in M^{\prime }}{\sum }R_{g,s},$$ (3)

where $M^{\prime }$ denotes the subset of module genes retained after identifier mapping and duplicate-gene handling. All retained genes contributed equally with weight $1/|M^{\prime }|$ ; if $|M^{\prime }|=0$ , the module score was set to missing. Composite programme scores were defined as unweighted means of their constituent module scores, for programme A with modules ${\left\{M_{k}\right\}}_{k=1}^{K}$ as shown in Equation 4:

$${COMP}_{A}\left(s\right)=\frac{1}{K^{\prime }}\underset{k\in K^{\prime }}{\sum }Score\left(M_{k},s\right),$$ (4)

where $K^{\prime }$ indexes modules available in the dataset $\left(K^{\prime }=|K^{\prime }|\right)$ , each contributing equally. Composite scores are interpreted as descriptive summaries of coordinated module activity rather than latent variables inferred via dimensionality reduction. Throughout, we report composite scores for stress response, myogenic progression cell-cycle activity ( ${\text{COMP}}_{\text{CELLCYCLE}}$ ), and environmental support ( ${\text{COMP}}_{\text{ITS}}$ ). Interpretation focuses on within-dataset relative differences and temporal patterns rather than absolute cross-platform magnitudes.

### 2.5 Gene-module definitions

Gene modules were curated *a priori* from established literature rather than inferred from the analysed datasets. Modules represent four conceptually distinct but biologically interacting dimensions: environmental support, stress signalling, cell-cycle state, and myogenic progression (Herschman, 1991; Bahrami and Drabløs, 2016).

Environmental support was represented by three axes that buffer metabolic/oxidative stress and permit myogenic progression: insulin/IGF signalling (AXIS IGF INSULIN; *IGF1R*, *INSR*, *IRS1*, *IRS2*, *PIK3CA*, *PIK3R1*, *AKT1*, *MTOR*, *RPTOR*, *FOXO1*) (Florini et al., 1996; Rommel et al., 1999); iron metabolism (AXIS IRON; *TFRC*, *TF*, *FTH1*, *FTL*, *SLC40A1*, *SLC11A2*, *ACO1*, *IREB2*, *HMOX1*, *SLC25A37*) (Wang and Pantopoulos, 2011); and redox/selenium homeostasis (AXIS REDOX SELENIUM; *SELENOP*, *GPX1*, *GPX4*, *TXNRD1*, *TXN*, *SOD1*, *SOD2*, *CAT*, *PRDX1*, *NQO1*) (Brigelius-Flohé and Flohé, 2020; Sies et al., 2017). Together these define ${\text{COMP}}_{\text{ITS}}$ .

Stress-associated transcription was represented by immediate-early genes (STRESS IMMEDIATE EARLY; *FOS*, *JUN*, *ATF3*, *EGR1*, *EGR2*, *IER2*, *DUSP1*) (Herschman, 1991; Bahrami and Drabløs, 2016), unfolded-protein-response genes (STRESS UPR ER; *DDIT3*, *XBP1*, *HSPA5*, *ATF4*, *PPP1R15A*, *DNAJB9*, *HERPUD1*), and integrated oxidative-stress genes (STRESS ISR OXIDATIVE; *HMOX1*, *SLC7A11*, *NFE2L2*, *GCLC*, *GCLM*, *TXNRD1*, *NQO1*, *ATF4*) (Pakos-Zebrucka et al., 2016; Wek, 2018; Costa-Mattioli and Walter, 2020), apoptotic-response genes (STRESS APOPTOSIS; *BAX*, *BBC3*, *PMAIP1*, *CASP3*, *CASP7*, *FAS*) (Fernando et al., 2002), and autophagy-related genes (STRESS AUTOPHAGY; *BECN1*, *ATG5*, *ATG7*, *MAP1LC3B*, *SQSTM1*) (Fortini et al., 2016). These modules define ${\text{COMP}}_{\text{STRESS}}$ .

Cell-cycle activity (STATE CELL CYCLE; *MKI67*, *TOP2A*, *CDK1*, *CCNB1*, *CCNB2*, *CCNA2*, *MCM2*, *MCM5*, *PCNA*, *TYMS*) defines ${\text{COMP}}_{\text{CELLCYCLE}}$ and captures proliferative state during commitment (Andrés and Walsh, 1996; Sabourin and Rudnicki, 2000).

Myogenic progression was represented by a stemness module (MYOGENIC STEMNESS; *PAX7*, *VCAM1*, *ITGA7*, *SPRY1*, *CXCR4*) and a commitment/differentiation module (MYOGENIC COMMITMENT; *MYOD1*, *MYF5*, *MYOG*, *MEF2C*, *MEF2D*, *MYH3*, *DES*, *TNNT1*, *ACTA1*). The latter defines ${\text{COMP}}_{\text{MYOGENIC}}$ (Weintraub et al., 1991; Zanou and Gailly, 2013).

#### 2.5.1 Module ambiguity and sensitivity analyses

Several genes participate in both environmental-homeostasis and stress-responsive pathways (for example, *HMOX1*, *TXNRD1*, *NQO1*, and *ATF4*) (Brigelius-Flohé and Flohé, 2020; Pakos-Zebrucka et al., 2016; Wek, 2018). To minimise circular interpretation, we prespecified shared genes and repeated analyses under alternative module definitions: (i) removal of shared genes from all modules, (ii) restriction of stress modules to immediate-early genes only, and (iii) restriction of environmental-support modules to selenium transport and antioxidant enzymes while excluding canonical ISR targets. We report whether qualitative separation between early stress-dominant states and coordinated myogenic progression persists across these specifications.

### 2.6 Operational indices for stress-dominant states and permissive differentiation modes

To separate stress-contingent transcriptional responses from environment-permitted myogenic progression, we defined two operational indices: Stress Response Index (SRI) and Permissive Differentiation Index (PDI). These quantities summarise relative programme dominance and alignment using scale-consistent transformations.

For each sample, ${\text{COMP}}_{\text{STRESS}}$ , ${\text{COMP}}_{\text{MYOGENIC}}$ , and ${\text{COMP}}_{\text{CELLCYCLE}}$ were computed as described above. In time-course datasets, replicate scores were averaged within each time point.

Cell-cycle exit was defined as shown in Equation 5:

$$Exit\left(t\right)=1-{COMP}_{\text{CELLCYCLE}}\left(t\right).$$ (5)

#### 2.6.1 Permissive alignment

Rather than estimating exit–lineage coupling by correlation (which is unstable for small n and undefined for non-temporal contrasts), we quantified alignment directly as the product of exit and myogenic progression as shown in Equation 6:

$$PDI=A=\bar{Exit}\;\cdot \;\bar{{COMP}_{\text{MYOGENIC}}},$$ (6)

where overbars denote means across time points (or samples within a condition). This definition is high only when cell-cycle withdrawal and myogenic activation are jointly elevated.

#### 2.6.2 Stress dominance

Stress dominance was expressed as a log-transformed ratio as shown in Equation 7:

$$\log R=\log\left(\frac{\bar{{COMP}_{\text{STRESS}}}}{\bar{{COMP}_{\text{MYOGENIC}}}+\epsilon }\right),$$ (7)

with $\epsilon =10^{-6}$ for numerical stability. Log transformation improves scale symmetry and reduces sensitivity to extreme ratios.

#### 2.6.3 Abruptness

For time-course datasets, abruptness of stress-associated dynamics was quantified using a normalised first-difference metric based on the median absolute successive change as shown in Equation 8:

$${Abrupt}_{\text{norm}}=\frac{{median}_{t}\left|\Delta {COMP}_{\text{STRESS}}\left(t\right)\right|}{IQR\;\left({COMP}_{\text{STRESS}}\left(t\right)\right)+\epsilon },$$ (8)

where Δ denotes successive differences and IQR is the interquartile range across time points. This provides a dimensionless summary that is less sensitive to any single time-step jump. For non-time-course two-group comparisons, ${Abrupt}_{\text{norm}}$ was set to zero.

#### 2.6.4 Stress response index

The revised stress index combines dominance, abruptness, and alignment as shown in Equation 9:

$$SRI=\log R+{Abrupt}_{\text{norm}}-A.$$ (9)

Higher SRI denotes states in which stress exceeds myogenic activation, stress dynamics are abrupt, and exit–myogenic alignment is weak. Lower SRI denotes more permissive differentiation modes with stronger alignment and limited abrupt stress contribution.

All indices were computed within datasets without cross-study scaling. For non-time-course contrasts (for example, wild-type versus ${Myod1}^{-/-}$ ), indices were calculated from group means with ${Abrupt}_{\text{norm}}=0$ .

#### 2.6.5 Weight sensitivity analysis

To assess robustness of composite ranking, we performed a three-dimensional grid search over weight parameters as shown in Equation 10:

$$w_{1},w_{2},w_{3}\in \left\{0.5,0.75,1.0,1.25,1.5\right\},$$ (10)

yielding 125 weight combinations. For each combination, conditions were ranked by SRI and stability was evaluated using (i) exact full-order stability, (ii) top- k and bottom- k stability (k=3) , and (iii) pairwise rank concordance via a Kendall-like coefficient as shown in Equation 11:

$$\tau =2\cdot \frac{\#\text{concordant pairs}}{\#\text{total pairs}}-1.$$ (11)

We additionally computed per-condition rank variance across weight combinations to identify conditions most sensitive to weight perturbation.

#### 2.6.6 Aggregation and parameter robustness analysis

To assess whether the main conclusions depended on the group-level averaging rule or the numerical stabiliser, we recomputed SRI and PDI using both mean- and median-based aggregation for stress, myogenic, and exit terms while retaining the definition of abruptness (median absolute first difference divided by the stress IQR), and we repeated these calculations across $\epsilon \in \left\{10^{-8},10^{-6},10^{-4},10^{-2}\right\}$ .

### 2.7 Statistical analysis

When raw integer counts were available, confirmatory differential-expression analyses used standard Bioconductor workflows (limma–voom and edgeR) via rpy2 (Ritchie et al., 2015; Robinson et al., 2010). Count matrices were wrapped in an edgeR DGEList; library-size normalisation used calcNormFactors (TMM); mean–variance modelling used voom. Multiple testing was controlled by the Benjamini–Hochberg false discovery rate (FDR) procedure (Benjamini and Hochberg, 1995). Datasets with only non-integer processed matrices were analysed descriptively. Principal-component analysis of TMM log-CPM values was used to assess within-dataset structure and trajectory geometry. In the Python-based downstream analyses, scikit-learn was used for principal-component analysis (sklearn.decomposition.PCA, two components for visualisation), and statsmodels was used for Benjamini–Hochberg FDR adjustment (statsmodels.stats.multitest.multipletests, method fdr_bh) (Seabold and Perktold, 2010; Pedregosa et al., 2011). Where descriptive two-group comparisons were reported from processed matrices or composite scores, we used two-sided Welch’s *t*-tests (scipy.stats.ttest_ind, equal_var=False) followed by FDR correction. Meanwhile, we additionally performed exact label-permutation tests for the observed mean differences. With n=3 versus n=3 , the exact two-sided permutation *p* value is bounded below by 0.10 because only 20 distinct allocations are possible; we therefore used these tests primarily to assess direction-consistent extremeness under minimal assumptions rather than to replace the descriptive effect estimates. We also carried out leave-one-out sensitivity analyses in which one replicate from each group was omitted in turn, and the resulting contrast was recomputed across all 3×3=9 reduced comparisons.

Because this study reanalyses public datasets generated across different platforms and experiments, robustness was handled at the analysis-design level. We applied one harmonised processing workflow per dataset (consistent gene-column handling, numeric sample filtering, duplicate-gene aggregation, and the same rank-based module/composite scoring) and restricted primary interpretation to within-study contrasts. Where biological replicates were available, scoring was performed at the sample level and then summarised at condition/timepoint level for trajectory metrics. Therefore, composite axes are interpreted as descriptive within-study summaries rather than as absolutely comparable magnitudes across studies.

## 3 Results

Results are presented in seven stages: (i) establish the primary serum-free bovine baseline, (ii) evaluate trajectory-level concordance in an independent serum-free single-nucleus dataset, (iii) assess how abrupt withdrawal reshapes early coordination in a bovine time-course, (iv) ask whether the withdrawal-associated pattern recurs in canonical murine C2C12 differentiation, (v) examine endpoint-level support from an independent enhanced-medium bovine dataset, (vi) test lineage dependence using a *Myod1* perturbation, and (vii) integrate the primary conditions on shared descriptive axes.

### 3.1 Serum-free differentiation and coordinated myogenic progression

**FIGURE 1:**
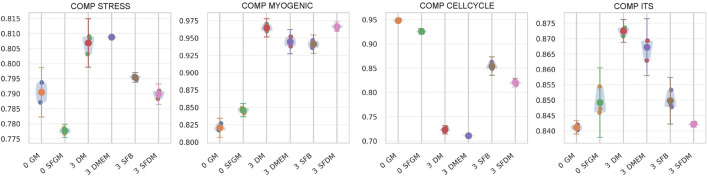
Serum-free bovine satellite-cell differentiation (GSE173196) supports myogenic progression without stress amplification. Composite programme scores summarise stress ( ${\text{COMP}}_{\text{STRESS}}$ ), myogenic progression ( ${\text{COMP}}_{\text{MYOGENIC}}$ ), cell-cycle activity ( ${\text{COMP}}_{\text{CELLCYCLE}}$ ) and environmental support ( ${\text{COMP}}_{\text{ITS}}$ ). GM, growth medium; DM, differentiation medium induced by serum withdrawal; SFGM, serum-free growth medium; SFDM, serum-free differentiation medium.

To establish a reference trajectory with minimal acute environmental perturbation, we first analysed bovine satellite-cell differentiation under serum-free conditions (GSE173196). We summarised each state using four rank-based composite axes—myogenic progression ( ${\text{COMP}}_{\text{MYOGENIC}}$ ), stress-associated transcription ( ${\text{COMP}}_{\text{STRESS}}$ ), proliferative activity ( ${\text{COMP}}_{\text{CELLCYCLE}}$ ), and environmental support ( ${\text{COMP}}_{\text{ITS}}$ )—allowing direct comparison of programme reweighting during induction (Figure 1; Table 2).

**TABLE 2:** Composite programme-score contrasts for bovine satellite-cell differentiation in GSE173196. Δ denotes the difference in mean composite score (B–A); 95% CIs are reported for Δ . Effect sizes are reported as Cohen’s d (standardised mean difference, $d=\Delta /s_{p}$ ). Note that for bounded rank-based composite scores in [0,1] and small n (here $n_{A}=n_{B}=3$ ), within-group variance can be extremely small, making $s_{p}$ tiny and inflating |d| (sometimes to tens) even when Δ is modest; therefore interpret d qualitatively and prioritise Δ and its CI. Multiple testing was controlled using Benjamini–Hochberg FDR. Numerical values are rounded to three decimal places.

Contrast	Axis	$n_{A}$	$n_{B}$	Δ (B–A)	95% CI	Cohen’s d	FDR
$0_{\mathrm{SFGM}}$ vs. $3_{\mathrm{SFDM}}$	${\text{COMP}}_{\text{STRESS}}$	3	3	0.012	[0.010, 0.015]	10.456	$5.39\times 10^{-4}$
$0_{\mathrm{SFGM}}$ vs. $3_{\mathrm{SFDM}}$	${\text{COMP}}_{\text{MYOGENIC}}$	3	3	0.126	[0.117, 0.134]	36.406	$1.57\times 10^{-5}$
$0_{\mathrm{SFGM}}$ vs. $3_{\mathrm{SFDM}}$	${\text{COMP}}_{\text{CELLCYCLE}}$	3	3	−0.110	[−0.118, −0.102]	−34.898	$1.76\times 10^{-5}$
$0_{\mathrm{SFGM}}$ vs. $3_{\mathrm{SFDM}}$	${\text{COMP}}_{\text{ITS}}$	3	3	−0.009	[−0.020, 0.003]	−2.682	$8.11\times 10^{-2}$
$0_{GM}$ vs. $3_{DM}$	${\text{COMP}}_{\text{STRESS}}$	3	3	0.014	[0.007, 0.022]	4.278	$6.34\times 10^{-3}$
$0_{GM}$ vs. $3_{DM}$	${\text{COMP}}_{\text{MYOGENIC}}$	3	3	0.150	[0.137, 0.163]	26.765	$1.04\times 10^{-5}$
$0_{GM}$ vs. $3_{DM}$	${\text{COMP}}_{\text{CELLCYCLE}}$	3	3	−0.234	[−0.242, −0.227]	−83.247	$1.04\times 10^{-5}$
$0_{GM}$ vs. $3_{DM}$	${\text{COMP}}_{\text{ITS}}$	3	3	0.033	[0.029, 0.037]	22.704	$1.45\times 10^{-4}$

Across the serum-free growth-to-differentiation transition ( $0_{\mathrm{SFGM}}$ to $3_{\mathrm{SFDM}}$ ), the dominant pattern was coordinated lineage progression: ${\text{COMP}}_{\text{MYOGENIC}}$ increased substantially ( Δ=0.126 , 95% CI [0.117, 0.134], FDR $=1.57\times 10^{-5}$ ), while ${\text{COMP}}_{\text{CELLCYCLE}}$ decreased in parallel ( Δ=−0.110 , 95% CI [-0.118, −0.102], FDR $=1.76\times 10^{-5}$ ). By contrast, ${\text{COMP}}_{\text{STRESS}}$ increased only modestly ( Δ=0.012 , 95% CI [0.010, 0.015], FDR $=5.39\times 10^{-4}$ ), and ${\text{COMP}}_{\text{ITS}}$ showed no strong depletion ( Δ=−0.009 , 95% CI [-0.020, 0.003], FDR $=8.11\times 10^{-2}$ ). Thus, under serum-free induction, transcriptional movement is dominated by reciprocal lineage gain and proliferative disengagement, rather than stress amplification.

As the contrast is based on n=3 biological replicates per group, we explicitly tested whether the directional conclusion depended on parametric assumptions or any single sample. Exact permutation tests showed that the observed shifts in ${\text{COMP}}_{\text{MYOGENIC}}$ , ${\text{COMP}}_{\text{STRESS}}$ , ${\text{COMP}}_{\text{CELLCYCLE}}$ , and ${\text{COMP}}_{\text{ITS}}$ were all at the directional extreme of the 20 possible label allocations (two-sided exact permutation *p* = 0.10; one-sided *p* = 0.05 for the observed direction in each case). Leave-one-out analysis gave the same result quantitatively: across all nine reduced comparisons, ${\text{COMP}}_{\text{MYOGENIC}}$ remained higher in $3_{\mathrm{SFDM}}$ than in $0_{\mathrm{SFGM}}$ ( Δ=0.122 –0.129), ${\text{COMP}}_{\text{CELLCYCLE}}$ remained lower ( Δ=−0.113 to −0.107 ), ${\text{COMP}}_{\text{STRESS}}$ remained only modestly elevated ( Δ=0.011 –0.013), and ${\text{COMP}}_{\text{ITS}}$ remained slightly lower ( Δ=−0.011 to −0.006 ). Thus, although the small sample size warrants caution, the main serum-free progression pattern is not driven by a single replicate or by the normal-theory assumptions of the Welch test.

As a within-dataset reference, we also compared the matched conventional serum-containing condition labels provided in the same study ( $0_{GM}$ to $3_{DM}$ ). This comparison showed the same directional pattern, but with larger magnitude shifts, including a stronger decline in ${\text{COMP}}_{\text{CELLCYCLE}}$ ( Δ=−0.234 , 95% CI [-0.242, −0.227], FDR $=1.04\times 10^{-5}$ ) and a moderate increase in ${\text{COMP}}_{\text{STRESS}}$ ( Δ=0.014 , 95% CI [0.007, 0.022], FDR $=6.34\times 10^{-3}$ ). Importantly, ${\text{COMP}}_{\text{ITS}}$ increased in this contrast ( Δ=0.033 , 95% CI [0.029, 0.037], FDR $=1.45\times 10^{-4}$ ), consistent with the notion that support-associated composite signal, as summarised by ${\text{COMP}}_{\text{ITS}}$ , is not obligatorily lost when differentiation programmes intensify.

**FIGURE 2:**
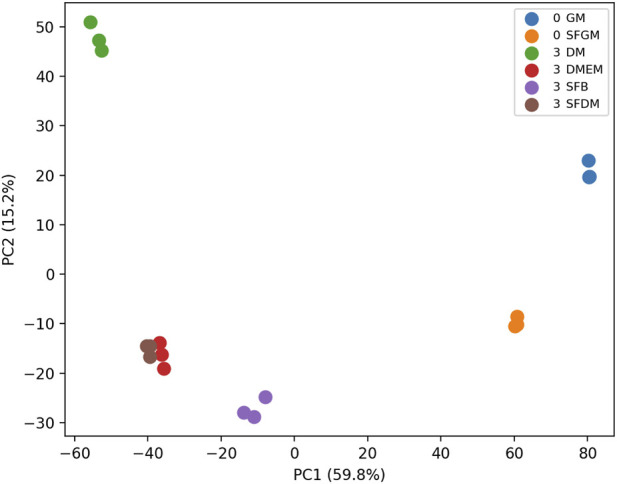
Principal-component analysis (PCA) of the bovine satellite-cell dataset GSE173196. PC1 (59.8%) primarily separates baseline growth conditions from day-3 states, whereas PC2 (15.2%) further resolves medium-context differences within the dataset. In particular, the conventional day-3 condition $3_{DM}$ is separated from the serum-free/defined day-3 states along PC2, consistent with an additional medium-dependent component of global transcriptomic variation beyond differentiation status alone.

Transcriptome-wide sample separation and coordinate shifts supported the same interpretation. PCA showed that PC1 (59.8%) primarily separated undifferentiated baseline growth conditions from day-3 differentiated states (Figure 2), consistent with a global transition from growth to differentiation at the transcriptome level. PC2 (15.2%) further separated cells cultured in conventional serum-containing media (GM and DM) from those cultured under serum-free or defined conditions. Notably, the conventional day-3 condition $3_{DM}$ was displaced from the serum-free/defined day-3 states along PC2, whereas $3_{\mathrm{SFDM}}$ remained closer to the broader non-withdrawal configuration. The auxiliary day-3 states $3_{\mathrm{DMEM}}$ and $3_{\mathrm{SFB}}$ are included here only as within-dataset contextual reference states rather than as primary contrasts. This pattern indicates that medium composition contributes an additional axis of transcriptomic variation beyond differentiation status alone. At the same time, the principal separation did not require a disproportionate expansion of stress-associated scores, reinforcing the inference that this non-withdrawal condition captures a comparatively ordered transition into myogenic state space.

### 3.2 Independent serum-free trajectory support in single-nucleus data

**FIGURE 3:**
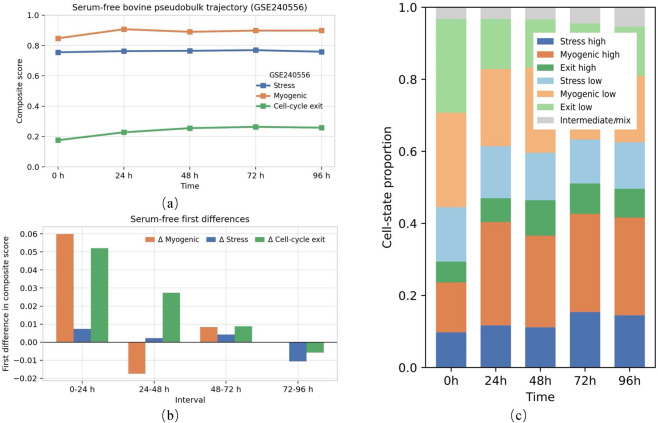
Independent serum-free bovine single-nucleus time-course (GSE240556). **(a)** Composite trajectories for stress, myogenic progression and cell-cycle exit across the pseudobulk serum-free trajectory. **(b)** First differences between consecutive time points in the same composite axes. **(c)** State-composition summary using mutually exclusive high/low classes, showing redistribution toward myogenic-high states without selective expansion of a stress-high population.

To assess whether the serum-free organisation observed in GSE173196 was specific to that accession, we analysed an independent bovine serum-free time-course profiled at single-nucleus resolution (GSE240556). Nuclei were aggregated by time point to generate pseudobulk profiles and scored with the same rank-based composite framework. Across the 0–96 h trajectory, ${\text{COMP}}_{\text{MYOGENIC}}$ increased from 0.847 to 0.898 and Exit increased from 0.176 to 0.258, whereas ${\text{COMP}}_{\text{STRESS}}$ remained confined to a comparatively narrow interval (0.755–0.769; Table 3 and Figure 3). These data provide independent trajectory-level support for the interpretation that serum-free progression can be accompanied by increasing myogenic organisation and proliferative disengagement without a marked expansion of the stress-associated composite axis.

**TABLE 3:** Independent serum-free bovine single-nucleus time-course (GSE240556) analysed as pseudobulk profiles by time point.

Time point	${COMP}_{\text{MYOGENIC}}$	Exit	${COMP}_{\text{STRESS}}$	${COMP}_{\text{ITS}}$
0 h	0.847	0.176	0.755	0.811
24 h	0.907	0.228	0.763	0.812
48 h	0.890	0.255	0.765	0.822
72 h	0.898	0.264	0.769	0.825
96 h	0.898	0.258	0.759	0.825

At single-nucleus resolution, this pattern appeared as a redistribution across descriptive high/low state classes rather than selective accumulation of a stress-only population, as shown in Figure 3c. Myogenic-high nuclei increased from 13.8% at 0 h to 27.2% at 96 h, while myogenic-low nuclei declined from 26.2% to 18.4% and exit-low nuclei declined from 26.0% to 13.7%. By contrast, the stress-high state remained a minority of nuclei across the time-course (9.8%–15.4%) and did not become the dominant late state. We therefore treat GSE240556 as an external serum-free trajectory check that belongs with the serum-free baseline logic, rather than as a generic validation result pooled with endpoint medium comparisons.

### 3.3 Acute serum withdrawal and transient lineage–stress misalignment

We next asked how the coordinated serum-free baseline is altered when differentiation is triggered by abrupt trophic withdrawal. To address this, we analysed a bovine withdrawal time-course (GSE173198) using the same composite framework, enabling direct comparison of temporal programme reweighting under matched scoring assumptions.

**FIGURE 4:**
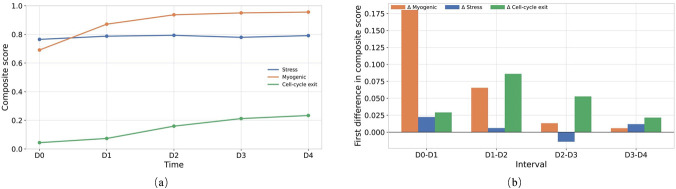
Bovine serum-withdrawal dynamics in GSE173198. **(a)** Composite trajectories for ${\text{COMP}}_{\text{STRESS}}$ , ${\text{COMP}}_{\text{MYOGENIC}}$ , and cell-cycle exit across the D0–D4 withdrawal time-course. **(b)** First differences (abruptness proxy) showing interval-to-interval changes in the same composite axes.

Following the medium switch, ${\text{COMP}}_{\text{STRESS}}$ and ${\text{COMP}}_{\text{MYOGENIC}}$ both increased (Figure 4a), indicating that withdrawal initiates a composite early response rather than a purely lineage-restricted transition. First-difference analysis showed that the immediate D0–D1 shift included a large myogenic gain, whereas cell-cycle exit increased more gradually across later intervals (Figure 4b). In descriptive terms, the withdrawal trajectory starts from a stress-weighted early state and then undergoes rapid lineage-associated reweighting, rather than following a purely lineage-restricted transition from the outset.

This temporal organisation has an important consequence for interpretation of early post-induction windows. Although lineage-associated activation is already detectable, early withdrawal samples retain high stress-associated weighting while exit-associated structure consolidates later, consistent with a short-lived misalignment between programme classes during entry into differentiation.

After the initial transition, cell-cycle exit became more pronounced, indicating delayed consolidation of exit-associated structure relative to the earliest composite response. Together, these dynamics support a model of partial early decoupling between stress, exit, and myogenic axes under withdrawal induction. This withdrawal-specific temporal offset provides the basis for the cross-condition comparisons developed below using SRI and PDI.

### 3.4 Early partial decoupling during canonical C2C12 withdrawal differentiation

**FIGURE 5:**
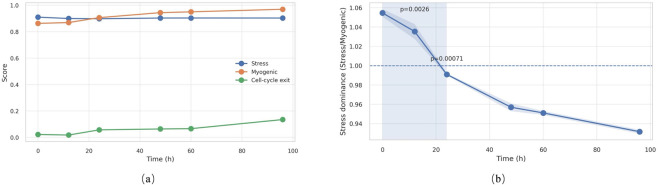
Canonical C2C12 differentiation by serum withdrawal (GSE224489) begins in a stress-dominant state. **(a)** Composite trajectories. **(b)** Stress-dominance dynamics with early post-switch elevation.

To determine whether the withdrawal-associated temporal offset observed in bovine cells also appears in canonical murine systems, we analysed a C2C12 serum-withdrawal time-course (GSE224489). Using the same composite axes, we quantified the balance between stress-associated and lineage-associated composite programme weighting through a stress dominance ratio over time (Table 4; Figure 5).

**TABLE 4:** Stress dominance ratio summary for the C2C12 serum-withdrawal time-course (GSE224489). Values report the mean stress dominance ratio with 95% CIs at each time point; p values correspond to the null hypothesis that the ratio equals 1. Numerical values are rounded to three decimal places.

Time (h)	n	Mean	95% CI (low)	95% CI (high)	p ( $H_{0}$ : ratio =1 )
0	3	1.055	1.050	1.059	$3.09\times 10^{-4}$
12	3	1.035	1.028	1.043	$2.61\times 10^{-3}$
24	3	0.991	0.990	0.992	$7.07\times 10^{-4}$
48	3	0.957	0.953	0.961	$5.00\times 10^{-4}$
60	3	0.951	0.949	0.953	$1.00\times 10^{-4}$
96	3	0.932	0.930	0.934	$4.86\times 10^{-5}$

At induction and the earliest post-switch interval, the ratio was greater than 1, indicating stress-dominant weighting (mean 1.055 at 0 h, 95% CI [1.050, 1.059]; mean 1.035 at 12 h, 95% CI [1.028, 1.043]). Thereafter, the ratio crossed below 1 and continued to decline (0.991 at 24 h, 0.957 at 48 h, 0.951 at 60 h, and 0.932 at 96 h), consistent with progressive relative enrichment of lineage-associated composite programme signal as differentiation proceeds. Thus, in this murine withdrawal model, the early phase is again characterised by transient stress predominance followed by gradual myogenic reweighting.

Trajectory-level behaviour supported the same interpretation. ${\text{COMP}}_{\text{MYOGENIC}}$ increased progressively across the time-course, whereas robust alignment between exit and lineage-associated components emerged later than the initial post-switch interval. This pattern is consistent with partial early decoupling followed by progressive realignment as cells move away from acute withdrawal entry dynamics.

Importantly, the directional structure matches that of the bovine withdrawal dataset—a stress-weighted early state transitioning towards stronger lineage weighting—supporting cross-species recurrence at the level of descriptive programme organisation. We therefore interpret these comparisons as evidence for conserved state-level behaviour, while avoiding claims of mechanistic identity between bovine satellite cells and murine C2C12 myoblast states.

### 3.5 Endpoint-level validation in an enhanced bovine medium dataset

Having already used GSE240556 as an independent serum-free trajectory check, we next asked whether endpoint-level bovine medium comparisons were directionally consistent with the same broad interpretation. This analysis was intended as complementary validation rather than as a replacement for the matched core comparison, because the dataset contains baseline and endpoint medium states rather than a continuous early differentiation time-course.

**FIGURE 6:**
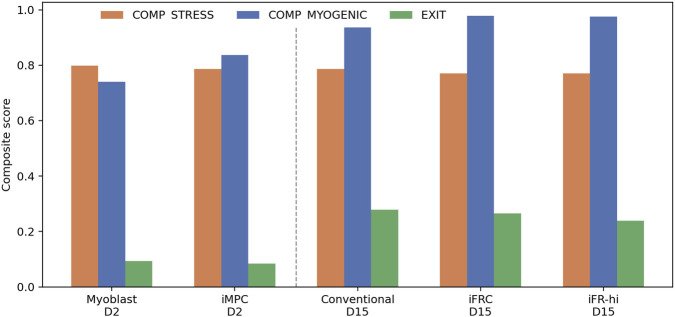
Endpoint-level bovine medium-state comparison in GSE262675. Myoblast D2 and iMPC D2 denote day-2 baseline proliferative media; Conventional D15 denotes day-15 conventional serum-withdrawal differentiation medium; ${\text{iFR}}^{hi}$ D15 denotes iMPC medium supplemented with high-dose forskolin and RepSox; and iFRC D15 denotes iMPC medium supplemented with forskolin, RepSox, and CHIR99021. Because GSE262675 contains baseline and endpoint medium states rather than a continuous transition, it was used only as complementary endpoint-level validation.

GSE262675 provided endpoint-level evidence from an enhanced myogenic differentiation system (Figure 6). This study compared day-2 baseline bovine myoblast cultures maintained in either conventional myoblast medium or iMPC medium with day-15 differentiated cultures exposed to conventional serum-withdrawal differentiation medium, ${\text{iFR}}^{hi}$ , or iFRC. In the source study, iMPC medium denotes a serum/serum-replacement medium containing bFGF; ${\text{iFR}}^{hi}$ denotes iMPC medium supplemented with high-dose forskolin and RepSox, whereas iFRC denotes iMPC medium supplemented with forskolin, RepSox, and CHIR99021. Because these samples represent baseline and endpoint medium states rather than a continuous differentiation time-course, we used GSE262675 only as a complementary validation dataset and did not interpret it as evidence for early transition kinetics. Within this endpoint framework, both enhanced differentiation conditions showed high ${\text{COMP}}_{\text{MYOGENIC}}$ scores at day 15 (iFRC, 0.978; ${\text{iFR}}^{hi}$ , 0.976), together with lower ${\text{COMP}}_{\text{STRESS}}$ than the conventional day-15 condition (0.771 and 0.770 versus 0.787). The day-2 baseline states were retained as reference states rather than interpreted as differentiation-entry states. In particular, the Myoblast D2 state showed a stress/myogenic ratio greater than 1 because ${\text{COMP}}_{\text{MYOGENIC}}$ was comparatively low in the baseline proliferative condition, not because this sample represented a withdrawal-like stress-dominant differentiation state. Thus, GSE262675 supports the limited conclusion that enhanced bovine differentiation media can achieve strong myogenic composite organisation without a corresponding increase in stress-associated weighting, but it does not substitute for matched early time-course comparisons.

### 3.6 *Myod1* ablation and dissociation of lineage and stress-associated programmes

**FIGURE 7:**
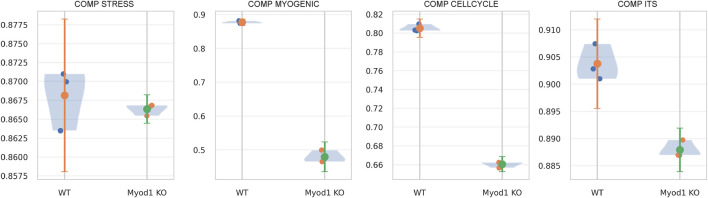
*Myod1* loss-of-function in serum-free C2C12 cells (GSE137507) reduces myogenic composite scores and shifts cell-cycle programme scores without altering stress-associated transcription.

Given that GSE137507 reflects a serum-free, non-canonical condition with reported neural-like features, this analysis should not be interpreted as evidence of myogenic differentiation dynamics. Instead, it provides a perturbational context in which canonical myogenic regulation is disrupted, allowing us to test whether stress-associated transcription can remain stable even when lineage identity is compromised. Within that restricted interpretation, *Myod1* loss reduced ${\text{COMP}}_{\text{MYOGENIC}}$ (NC mean 0.878 vs. NK mean 0.480; Δ=−0.398 , FDR $=7.85\times 10^{-4}$ ) and was associated with lower ${\text{COMP}}_{\text{CELLCYCLE}}$ (NC 0.805 vs. NK 0.661; Δ=−0.145 , FDR $=6.46\times 10^{-6}$ ), indicating attenuated lineage progression with a shifted cell-cycle programme (Table 5; Figure 7). By contrast, ${\text{COMP}}_{\text{STRESS}}$ was not detectably changed ( Δ=−0.002 , FDR =0.522 ). These data show that lineage-associated components are *Myod1*-dependent, whereas stress-associated composite scores can remain stable despite loss of this regulator. In this restricted perturbational sense, lineage and stress axes are empirically dissociable.

**TABLE 5:** Composite programme scores in wild-type (NC) versus ${Myod1}^{-/-}$ (NK) C2C12 cells (GSE137507). Δ denotes the difference in mean score (NK–NC). Multiple testing was controlled using Benjamini–Hochberg FDR. Numerical values are rounded to three decimal places.

Score	NC mean	NK mean	Δ (NK–NC)	p	FDR
${COMP}_{\text{CELLCYCLE}}$	0.805	0.661	−0.145	$1.61\times 10^{-6}$	$6.46\times 10^{-6}$
${COMP}_{\text{MYOGENIC}}$	0.878	0.480	−0.398	$3.92\times 10^{-4}$	$7.85\times 10^{-4}$
${COMP}_{\text{ITS}}$	0.904	0.888	−0.016	$5.67\times 10^{-3}$	$7.57\times 10^{-3}$
${COMP}_{\text{STRESS}}$	0.868	0.866	−0.002	$5.22\times 10^{-1}$	$5.22\times 10^{-1}$

### 3.7 Separation of permissive and withdrawal conditions along descriptive axes

After characterising each dataset separately, we integrated all conditions using the operational indices defined in Methods: SRI (stress dominance with abruptness and coupling penalty) and PDI (exit–myogenic alignment). Condition-level values are summarised in Table 6.

**TABLE 6:** Condition profiles under revised operational indices. PDI quantifies exit–myogenic alignment $\left(A=\bar{Exit}\cdot \bar{Myogenic}\right)$ . SRI integrates log stress dominance, normalised abruptness, and alignment.

Condition	Stress/Myogenic	${\text{Abrupt}}_{\text{norm}}$	PDI	SRI
*Bovine GSE173196 (serum-free and medium-context conditions)*				
$0_{GM}$	0.969	-	0.044	−0.075
$0_{\mathrm{SFGM}}$	0.920	-	0.065	−0.149
$3_{DM}$	0.833	-	0.278	−0.460
$3_{\mathrm{DMEM}}$	0.854	-	0.284	−0.442
$3_{\mathrm{SFB}}$	0.841	-	0.143	−0.315
$3_{\mathrm{SFDM}}$	0.813	-	0.181	−0.388
*Bovine GSE173198 (serum-withdrawal time-course)*				
Withdrawal	0.889	1.093	0.127	0.848
*Mouse GSE224489 (serum-withdrawal time-course)*				
Withdrawal	0.985	0.646	0.056	0.575
*Mouse GSE137507 (serum-free Myod1 perturbation)*				
Wild-type (NC)	0.989	-	0.171	−0.182
${Myod1}^{-/-}$ (NK)	1.806	-	0.163	0.429
*Bovine GSE262675 (grouped medium states)*				
Myoblast D2	1.079	-	0.069	0.007
iMPC D2	0.940	-	0.071	−0.133
Conventional D15	0.828	-	0.265	−0.454
iFRC D15	0.788	-	0.260	−0.499
${iFR}^{hi}$ D15	0.790	-	0.234	−0.470

Within bovine datasets, the GSE173196 serum-free and related medium-context conditions all remained in the negative-SRI range, with moderate-to-high PDI and zero abruptness, consistent with coordinated differentiation under non-withdrawal conditions. By contrast, bovine withdrawal (GSE173198) showed positive SRI (0.848), driven by high normalised abruptness (1.093) and weaker alignment (PDI = 0.127), despite stress/myogenic ratios in the same broad range as the GSE173196 conditions.

Murine analyses showed the same directional separation: C2C12 withdrawal (GSE224489) occupied the high-SRI, low-PDI region (SRI 0.575; PDI 0.056), whereas serum-free wild-type C2C12 was low-SRI and better aligned (SRI =−0.182 , PDI =0.171 ). ${Myod1}^{-/-}$ samples had positive SRI (0.429) despite zero abruptness, indicating that stress-to-lineage imbalance can also arise through compositional reweighting without an abrupt temporal transition.

The additional bovine analyses were directionally concordant with this separation but were not pooled into a generic validation category. GSE240556 was interpreted with the serum-free trajectory results because it tests the same progression logic at single-nucleus resolution, whereas GSE262675 was retained as grouped endpoint medium states because it does not measure early transition kinetics.

Across datasets, serum-withdrawal conditions were distinguished from non-withdrawal conditions by three recurrent features: greater early stress weighting, larger initial abruptness, and weaker early coupling between myogenic activation and cell-cycle exit. These features appeared in both bovine and murine withdrawal time-course datasets, whereas non-withdrawal conditions showed more gradual trajectories and stronger alignment. Taken together, these analyses support a profile-based interpretation in which induction protocols reweight programme components and alter their timing within a shared transcriptional space. We therefore use SRI and PDI as operational axes for protocol-level comparison rather than mechanistic variables.

Weight perturbation stability of ordering Across 125 weight combinations, exact full ordering was preserved in 55.2% of cases (69/125). Stratification was more stable: the top-3 high-SRI conditions were unchanged in 92.0% of combinations and the bottom-3 conditions in 67.2%. Pairwise concordance remained high (median Kendall-like τ=1.000 , range 0.778–1.000), indicating that weight perturbation primarily affected mid-spectrum ordering without altering the high-level separation between stress-dominant states and lineage-aligned states.

Robustness to aggregation rule and ϵ choice Using independently regenerated score matrices for sensitivity analysis, the key directional conclusions remained unchanged when SRI/PDI were recomputed with alternative group-level averaging rules and across $\epsilon \in \left\{10^{-8},10^{-6},10^{-4},10^{-2}\right\}$ . Across all tested specifications, bovine withdrawal remained higher in SRI than $3_{\mathrm{SFDM}}$ (withdrawal: 0.260–0.849; $3_{\mathrm{SFDM}}$ : −0.400 to −0.388), whereas $3_{\mathrm{SFDM}}$ remained higher in PDI than bovine withdrawal ( $3_{\mathrm{SFDM}}$ : 0.181–0.182; withdrawal: 0.127–0.149). Likewise, murine withdrawal remained higher in SRI than serum-free wild-type (0.052–0.576 versus −0.193 to −0.180), and ${Myod1}^{-/-}$ remained higher in SRI than wild-type (0.408–0.445 versus −0.193 to −0.180).

## 4 Discussion

Myogenic differentiation *in vitro* is often described as a unidirectional process that begins at serum withdrawal. Our reanalysis supports a more conservative interpretation: the induction step is itself an acute environmental perturbation, and early bulk transcriptional states can show increased stress-associated weighting together with weaker alignment between myogenic activation and cell-cycle exit. By comparing trajectories across induction conditions within each dataset, and then asking whether the same descriptive organisation reappears in independent bovine settings, we establish an operational framework that summarises condition-dependent composite transcriptional organisation.

In bovine satellite-cell data, serum-free differentiation followed a gradual transition in which myogenic activation tracked cell-cycle withdrawal, consistent with coordinated progression under those conditions. In the matched bovine serum-withdrawal time-course, abrupt serum reduction produced an early composite state marked by elevated stress-associated weighting and weaker exit–myogenic coupling. This pattern indicates that early post-switch samples can occupy a state in which stress-responsive programmes contribute substantially to the composite state, complicating use of early time points as direct proxies for durable commitment. Independent bovine analyses supported the same broader contrast at both bulk-like and single-nucleus levels: under comparatively stable induction conditions, progression was characterised by rising myogenic organisation, stronger exit-related structure, and redistribution toward myogenic-containing cell states without selective expansion of a stress-only population, whereas more conventional endpoint settings retained relatively higher stress weighting. These conclusions are based on within-study contrasts, and we do not assume quantitative equivalence of score magnitudes between bovine and murine systems.

The murine C2C12 withdrawal time-course showed the same qualitative composite-score pattern: stress dominance was highest in the earliest post-switch window and declined thereafter, while myogenic programmes increased over the time-course. Thus, the interval often labelled “early differentiation” can include a stress-enriched composite state even as canonical myogenic regulators begin to change. Practically, early marker shifts can therefore admit multiple, non-exclusive interpretations (Herschman, 1991; Bahrami and Drabløs, 2016): immediate-early and integrated-stress responses may be captured alongside transcriptional changes more tightly linked to commitment, with stronger coupling to cell-cycle exit emerging after the acute phase resolves (Andrés and Walsh, 1996; Shen et al., 2003; Ruijtenberg and van den Heuvel, 2016).

Genetic perturbation provides a within-condition dissociation between these axes. Under serum-free conditions, *Myod1* ablation strongly reduced the myogenic composite score and altered cell-cycle programmes, but left stress-associated transcription largely unchanged. However, this observation is derived from a non-canonical serum-free condition originally associated with neural-like state transitions, and therefore should be interpreted as an extreme perturbational scenario rather than a physiological differentiation context. In this restricted sense, stress-associated scores are not sufficient to recapitulate myogenic identity, and lineage-specific transcription factors remain primary determinants of the myogenic axis (Buckingham and Rigby, 2014; Bentzinger et al., 2012). More broadly, interventions that modulate stress pathways may alter early transcriptional readouts without moving cells along the same trajectory associated with durable myogenic progression (Herschman, 1991; Bahrami and Drabløs, 2016; Pakos-Zebrucka et al., 2016).

The robustness analyses reduce concern that the main conclusions are artefacts of a single aggregation rule or ϵ choice, because the separation between withdrawal and non-withdrawal conditions remained stable across all tested specifications. The added support from independent bovine datasets further argues that the qualitative interpretation is not confined to a single accession. At the same time, closely matched public datasets directly comparing serum-withdrawal with serum-free or defined myogenic induction remain limited, and the currently available validation sets differ in modality or endpoint structure; broader validation will therefore benefit from future datasets generated under more standardised medium-comparison designs. These results help explain discrepancies across the myogenesis literature. Studies focused on early windows, short perturbation intervals, or acute transcriptional responses may conflate composite states that differ in programme weighting, timing, and alignment across protocols. Our framework suggests that part of the variability in reported timelines and pathway dependencies may arise from differences in how strongly induction perturbs trophic support and stress-control systems at the moment of switching. In practical terms, serum withdrawal can create a transient, protocol-dependent early state whose readouts are not necessarily interchangeable with those from non-withdrawal conditions (Messmer et al., 2022).

The findings are also relevant to translational and bioprocessing contexts, with appropriate caution given the absence of new functional assays. In regenerative manufacturing and cultured-meat workflows, the key endpoint is durable acquisition of myogenic identity and maturation potential, whereas bulk transcriptional shifts during the first 24–72 h may reflect varying mixtures of lineage- and stress-associated programme weighting. Our results support *paired* quality-control readouts that combine canonical myogenic markers with stress-associated modules and exit-related measures to identify protocols showing elevated early stress weighting and weaker exit–myogenic alignment. Whether such composite readouts predict later maturation or function will require prospective validation using orthogonal assays (for example, fusion index, sarcomeric organisation, contractility, and long-term stability).

Several limitations are important. First, this is a reanalysis of public bulk RNA-seq data, and composite scoring necessarily compresses heterogeneous biology into low-dimensional summaries; single-cell and functional data will be needed to resolve whether stress dominance reflects compositional shifts, within-cell rewiring, or both. Second, several key two-group contrasts in GSE173196 involve only three biological replicates per group. Our added exact-permutation and leave-one-out analyses reduce concern that the main directional conclusions are artefacts of distributional assumptions or of any single replicate, but they do not substitute for future experimental replication with larger sample sizes. Third, dataset-specific factors (including serum source, confluence, substrate, and sampling cadence) may influence abruptness and coupling estimates, although recurrence of the same directional separation across species and perturbations supports generality. Fourth, the indices introduced here are operational rather than mechanistic; identifying causal environmental drivers of early state configuration will require controlled experiments that systematically vary growth-factor, hormone, lipid, and carrier-protein inputs.

Overall, the early period after serum withdrawal is frequently characterised by elevated stress-associated weighting and altered alignment with myogenic and cell-cycle-exit programmes relative to later stages. Explicitly summarising composite structure and state-dependent alignment provides a principled basis for protocol comparison and for interpretation of early differentiation readouts in both basic and applied myogenesis research.

## 5 Conclusion

Our reanalysis indicates that abrupt serum withdrawal, widely used to induce C2C12 differentiation, is associated with a transient early state enriched for stress-associated transcription rather than a purely lineage-driven transition. Across bovine and murine withdrawal time-course datasets, early post-switch samples showed greater stress dominance and weaker coupling between myogenic activation and cell-cycle exit than non-withdrawal conditions. Independent bovine validation datasets provided directionally consistent support for the same broad descriptive contrast under non-withdrawal conditions or enhanced media, without constituting fully matched validation of the primary comparisons. In a non-canonical serum-free *Myod1* perturbation, lineage-associated programmes were markedly reduced whereas stress-associated transcription remained similar, consistent with a restricted perturbational dissociation. Together, these findings support a cautious interpretive framework in which early bulk RNA-seq changes are interpreted as state-dependent reweighting and temporal offset of stress and lineage components, and they motivate joint evaluation of myogenic progression and stress dominance when comparing induction protocols and interpreting early differentiation readouts.

## Data Availability

The datasets analysed in this study are publicly available in the NCBI Gene Expression Omnibus (GEO) under accession numbers GSE173199 (superseries), GSE173196, GSE173198, GSE240556, GSE262675, GSE224489, and GSE137507. GSE173199 is the parent superseries for the two bovine subseries, while the additional validation accessions were used to test directional concordance of the framework across related bovine settings.
